# Systematic Analysis of Protein Interaction Network Associated with Azoospermia

**DOI:** 10.3390/ijms17111857

**Published:** 2016-11-10

**Authors:** Soudabeh Sabetian, Mohd Shahir Shamsir

**Affiliations:** Department of Biological and Health Sciences, Faculty of Bioscience & Medical Engineering, Universiti Teknologi Malaysia, 81310 Johor, Malaysia; shahir@utm.my

**Keywords:** azoospermia, gene ontology, infertility, protein interaction network

## Abstract

Non-obstructive azoospermia is a severe infertility factor. Currently, the etiology of this condition remains elusive with several possible molecular pathway disruptions identified in the post-meiotic spermatozoa. In the presented study, in order to identify all possible candidate genes associated with azoospermia and to map their relationship, we present the first protein-protein interaction network related to azoospermia and analyze the complex effects of the related genes systematically. Using Online Mendelian Inheritance in Man, the Human Protein Reference Database and Cytoscape, we created a novel network consisting of 209 protein nodes and 737 interactions. Mathematical analysis identified three proteins, ar, dazap2, and esr1, as hub nodes and a bottleneck protein within the network. We also identified new candidate genes, *CREBBP* and *BCAR1*, which may play a role in azoospermia. The gene ontology analysis suggests a genetic link between azoospermia and liver disease. The KEGG analysis also showed 45 statistically important pathways with 31 proteins associated with colorectal, pancreatic, chronic myeloid leukemia and prostate cancer. Two new genes and associated diseases are promising for further experimental validation.

## 1. Introduction

Male factor infertility is involved in over 50% of couples trying to conceive [1]. The increasing role of the male factor in cases of couples’ infertility has been identified due to the increased evaluation of male reproductive function and the development of new diagnostic tools [2,3]. Despite this, azoospermia is always taken as a matter of serious interest while treating the issues related to infertility [4]. Non-obstructive azoospermia (NOA) is considered to be a severe male infertility factor due to the impaired spermatogenesis with the consequent absence of spermatozoa in the ejaculate [5]. Although intracytoplasmic sperm injection (ICSI) as an assisted reproductive technology is an efficient therapy for severe male infertility, the success rate for NOA cases following ICSI therapy is around 36% [6]. Previous research has studied the medical treatment to improve the sperm quality of the patients before carrying out ICSI cycles [7]. Therefore, while understanding the overall mechanism of azoospermia is very important to improve the medical therapy, the underlying etiology and mechanism(s) remain elusive [4,7].

The presence of androgens is necessary for the production of normal sperm. It has been found that defective spermatogenesis is caused by lowering the levels of intratesticular androgens [8]. Recent research has established a new relationship of the androgen receptor gene with azoospermia [9] where most patients suffering from idiopathic azoospermia have normal levels of serum androgen, which indicates that the etiology of this condition might be due to the faults in the androgen response pathway [8]. Chromosome disorders are considered as the most common genetic defect in NOA [10].

In order to identify the candidate genes which are associated with NOA, several techniques have been adopted. It has been determined if the men suffering from azoospermia have an increased risk of cancer; however, most of the conducted research has not extensively taken the molecular biological pathways or the systematic interactions between the components into consideration [11,12,13]. In this study, construction and analysis of a protein-protein interaction network were applied in order to extensively consider all the cell functions of the proteins associated with the azoospermia and the molecular biological pathways of the genetic links between azoospermia and other diseases.

## 2. Results and Discussion

### 2.1. Construction and Parameters of Protein-Protein Interaction Protein-Protein Interaction (PPI Network)

**Figure 1:**
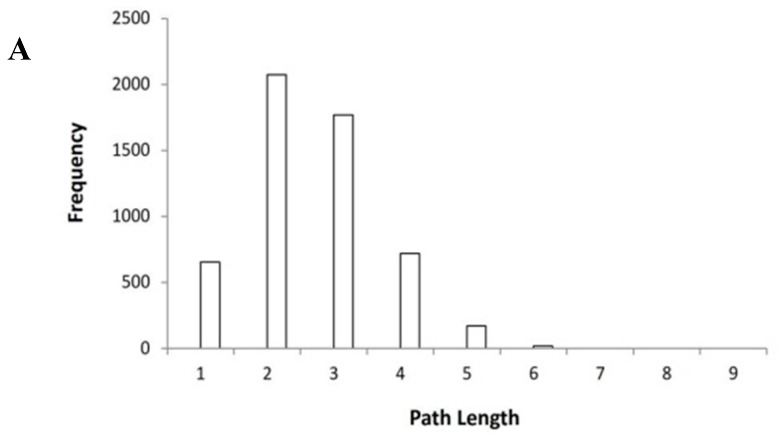

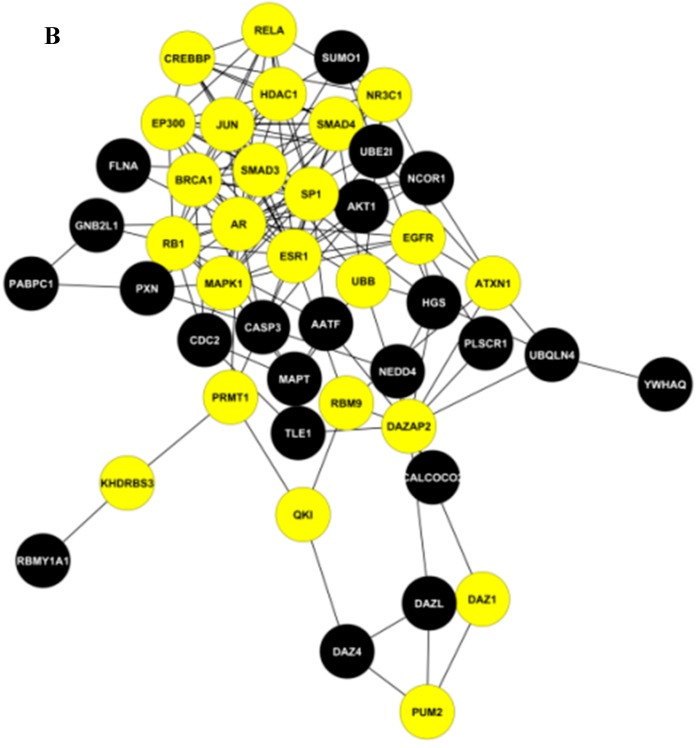
(**A**) Histogram showing distribution of the shortest path. Two randomly selected nodes were connected via 2.85 links; (**B**) The small (simplified) network of the Protein-Protein Interactions (PPIs) of azoospermia. The identified important nodes and their interconnected nodes were extracted to construct a new small PPI network. This new network consists of 46 nodes and 157 links. The key nodes are highlighted in yellow.

The initial selection of proteins from the Online Mendelian Inheritance in Man (OMIM) database produced 86 proteins associated with azoospermia (refer to supplementary file). Examination of the identified proteins in Protein-Protein Interaction (PPI) data from the Human Protein Reference Database (HPRD database) showed that there are 209 related proteins with 737 links (refer to the supplementary file). The retrieved data were entered into Cytoscape 2.8 to construct the PPI network. The R package was used to calculate the topological parameters. The mean degree value of the protein nodes is 7.05. The shortest path analysis of the network showed that any two randomly selected nodes on the network were connected via 2.85 links. The degree exponent value is lower than 3 [14], which is similar to the other networks following a scale-free distribution and similar to values of other human protein networks [15]. A histogram is used to plot the distribution of the shortest paths (Figure 1A).

### 2.2. The Key Nodes of the PPI Network

In a scale-free distribution, the presence of a small number of highly connected nodes known as hubs is more important than lesser connected nodes, which is one of the important features of networks [14,16]. The hub nodes play an important role in the survival of cells because if the hub nodes are attacked in a network, the network can be broken into pieces [17]. The nodes that possess a degree or a Betweenness Centrality (BC) value larger than the mean were selected (Table 1). Twenty-two of the nodes in the network are proteins that have been associated with azoospermia.

Examination of the key nodes identified in our PPI network suggested proteins crebbp and bcar1, which have never been associated with azoospermia. crebbp is responsible for handling Gap 1 (G1) arrest during the cell cycle [18] and bcar1 is responsible for the maintenance of the cell in the focal adhesion pathway [19]. bcar1 interacts with nephrocystin and both proteins localize to cell-cell contacts of polarized epithelial cells [20]. Nephrocystin is involved in spermatogenesis and is required for the differentiation of early elongating spermatids into spermatozoa [21]. Previously, it has been reported that knockdown CREBBP eliminated gnrh; thus, CREBBP may function to regulate the reproductive process [22]. The rest of the proteins identified as hub and key nodes are those previously associated with azoospermia. For example, the MAPK signaling pathway carries out the communication in cells and RB1 maintains the cycle of the cell [18]. For example, the activation of the MAPK cascade in a complex manner is required for the generation of mature spermatozoa in the epididymis [23]. MAPKS_regulate mature spermatozoa flagellar motility, hyperactivation and the acrosome reaction, and they are also involved in the regulation of transcription and ectoplasmic specialization (ES) in the testis [23]. rb1 plays an essential role in the foundation of the spermatogonial stem cell (SSC) pool during neonatal development [24].

Three proteins, the dazap2, androgens and a functional ar and esr1 had both a large BC value and degree. The first is the ar protein, which plays an important role in the maintenance and development of the male spermatogenesis and phenotype. The *AR* is comprised of eight exons and is determined by a gene located on the X chromosome. The process of transformation of the genes plays an important role in male infertility from a genetic point of view and is observed with a prevalence of about 2% in randomly selected infertile men [25]. The majority of the patients who suffer from idiopathic azoospermia show normal serum levels of androgens. It signifies the fact that the defects in the androgen response pathway might be responsible in the etiology of this condition. By binding to the androgen receptor, both testosterone and dihydrotestosterone show their biological activities. The possible cause of idiopathic azoospermia is thought to be due to the abnormalities in the AR, and for this reason it has been given great importance [26]. The second is dazap2, which is an interacting protein of germ-cell–specific RNA-binding proteins *DAZ* (deleted in azoospermia) [27]. The dazap2 protein plays a role as the interacting partner of tcf-4. The knockdown of *DAZAP2* does not only lessen the activity of Wnt signaling as measured by Tcf/-catenin reporters, but it also changes the expression of Wnt signaling target genes [28]. The affinity of tcf-4 is modulated by dazap2 for its DNA recognition role in chromatin immunoprecipitation studies. The reduced expression of hoxb9, while simultaneously increasing the front marker otx2, is the result of the reduction of dazap2 in embryos. *DAZAP2* is necessary for the FGF-dependent posterior patterning. In contrast to the FGF activity, dazap2’s introduction of hoxb9 is not congested by the loss of canonical Wnt signaling and the increment in the level of dazap2 changes neural modeling and induces posterior neural markers [29]. The molecular-based functions of daz and dazl proteins were observed by studying RNAs and proteins which come in contact with *DAZ* and *DAZL*. As reported in earlier studies, the representation of the association of the daz and dazl proteins with pum2, a human homolog of the pumilio gene in *Drosophila* that is required to preserve germ-line stem cells, plays an important role during embryogenesis and the development of the germ cell [30]. The third protein, esr1 is required to mediate the estrogen cellular effect in normal male reproduction and mutations in this gene may lead to spermatogenic disturbances [31]. Even the esr1 gene polymorphism has been associated with idiopathic non-obstructive azoospermia [32]. Polymorphism of these genes, *DAZAP2*, *AR* and *ESR1*, has been reported to be associated with male infertility [33].

The identified important nodes and their interconnected nodes were extracted to construct a new, small PPI network. This new network consists of 46 nodes and 157 links (Figure 1B).

### 2.3. Gene Ontology (GO) Enrichment Analysis

**Figure 2:**
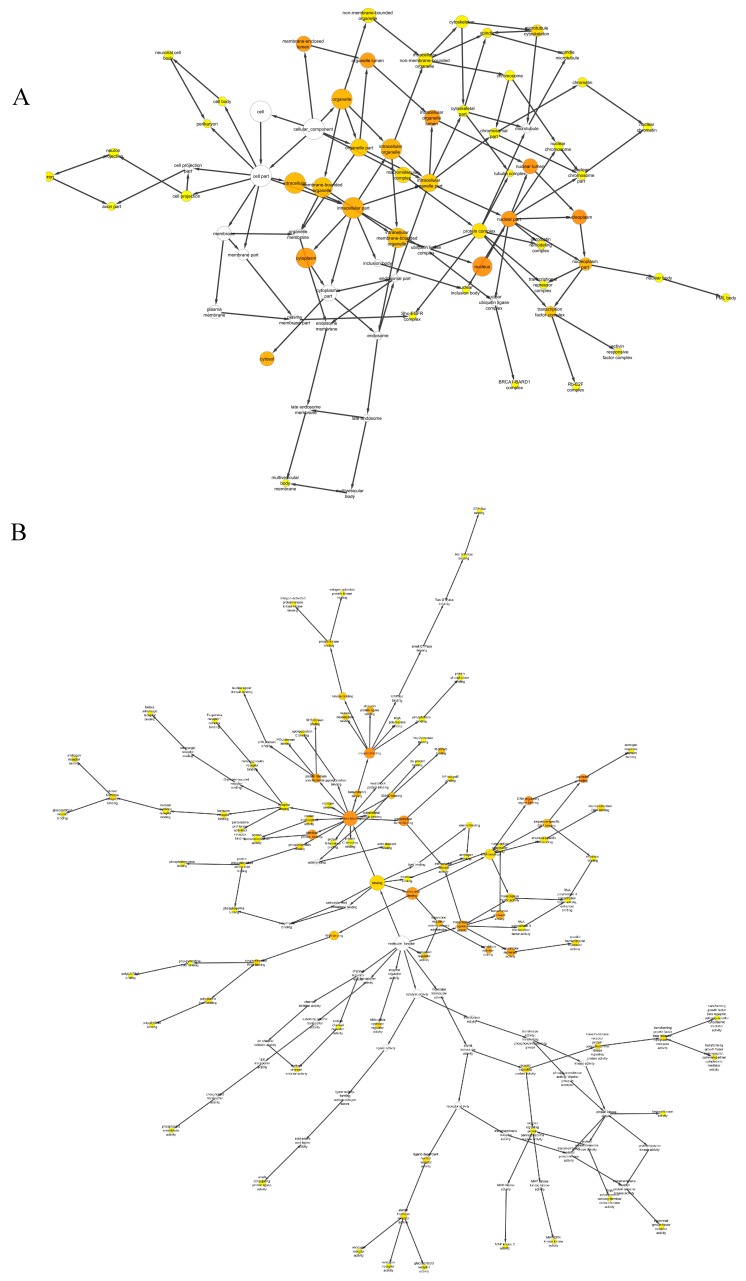
The BiNGO analysis regarding: cellular component and molecular function. (**A**) Map of the cellular component and molecular function associated with the azoospermia small network. Darker nodes refer to the substantial ontologies of the dataset. The majority of the proteins are in the nucleus, cytoplasm and intracellular part; (**B**) In terms of function and biological process, the majority of the proteins are involved in protein binding, and are thereby linked to other protein nodes such as protein domain–specific binding, identical protein binding, SMAD binding, identical protein binding, phosphoproteins binding, β-catenin binding and transcription factor binding.

The small network with the main pathways was then analyzed with the BiNGO and ClueGO programs to identify the major ontologies involved in the protein network, the cellular components, the molecular function and the biological process (Figure 2A,B). The result showed that the majority of the proteins are located in the nucleus, cytoplasm and intracellular spaces. In terms of function and biological process, the majorities of the proteins are involved in protein binding and are thereby linked to other protein nodes such as in protein domain-specific binding, identical protein binding, SMAD binding, phosphoproteins binding, β-catenin binding and transcription factor binding (Figure 2B). These proteins are related to the process of sperm production which requires many enzymes and transcription factors that act as switches, precisely regulating the expression of genes that in turn control the developmental program of male germ cells [34,35].

**Figure 3:**
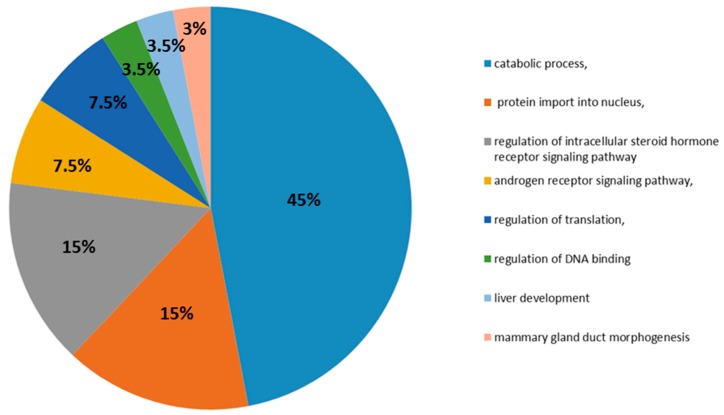
ClueGo analysis. In terms of biological processes, the top categories are regulation of the protein catabolic process, protein import into the nucleus, regulation of the intracellular steroid hormone receptor signaling pathway, regulation of the androgen receptor signaling pathway, regulation of translation, regulation of DNA binding, liver development and mammary gland duct morphogenesis.

In biological processes, the top ontological categories are the regulation of the protein catabolic process, protein import into the nucleus, regulation of the intracellular steroid hormone receptor signaling pathway, regulation of the androgen receptor signaling pathway, regulation of translation, positive regulation of translation, liver development, regulation of DNA binding and mammary gland duct morphogenesis (Figure 3).

These biological processes are important in sperm production and successful capacitation of spermatozoa [4,35]. We identified two unique ontologies: liver development and mammary gland duct morphogenesis. Five hub proteins including ep300, jun, rela, smad3 and sp1 have been identified in previous research on liver development. Loss of the normal male secondary characteristics is common in patients with liver disease [36]. Severe liver disease results in incomplete metabolism of the sex hormones and thus results in an accumulation of circulatory estrogen that suppresses gonadotropin secretion, leading to a drop in the sperm count, and possibly to azoospermia [37]. Our analysis shows that there is a possible genetic connection between liver disease and azoospermia.

### 2.4. DAVID (Database for Visualization, Annotation, and Integrated Discovery) Analysis

The KEGG analysis using DAVID functional annotation tools indicated that 28% of the proteins in the small network are involved in cancer pathways (refer to the supplementary file). These proteins are involved in the pathways of colorectal, pancreatic, chronic myeloid leukemia and prostate cancer. The analysis shows possible genetic links between azoospermia and cancers. Men with azoospermia have previously been linked with an increased risk of subsequently developing cancer, suggesting a possible common etiology between azoospermia and cancer development [38].

## 3. Materials and Methods

### 3.1. Selecting Associated Proteins with Azoospermia

The first step involves the selection of genes associated with azoospermia from the OMIM (available on: http://www.omim.org/), which has been found to be the most authentic and reliable source of information as compared to the published papers [39] using the keyword “azoospermia”. The identified genes are used as the basis for the construction of the PPI network.

### 3.2. Construction of the PPI Network

To construct the PPI network, we have identified the protein-protein interactions of the collected proteins using the HPRD (available on: http://www.hprd.org/) [40,41]. All retrieved data were loaded into Cytoscape 2.8 [42] to construct the protein interaction network (PIN).

### 3.3. Topology and Parameter Analysis of PPI Network

The target proteins that play important roles in the network were evaluated through the following measurements: (1) degree (or connectivity); (2) the BC; (3) shortest path; and (4) the closeness centrality (CC). The term degree is used to identify the number of links of a node to other nodes. The degree distribution indicates a relatively small number of highly connected nodes that are known as hubs, which play a major role in the network as a local property [14]. The measurement of the BC is very useful for the detection of bottlenecks in a network. For node *k* BC is defined as

$$b\left(k\right)=\;\sum_{\left(i,j\right)}bi\to j\left(k\right)=\sum_{\left(i,j\right)}\left(g_{{\left(i\to j\right)}^{k}}\right)/g_{\left(i\to j\right)}\;$$

where *g*_(*i*→*j*)_ is the number of shortest paths from node *i* to node *j*, and *g*_(*i*→*j*)*^k^*_ is the number of geodesics among *g*_(*i*→*j*)_ from node *i* to node *j* that pass through node *k* [43,44].

To check for bottlenecks in the networks, the BC value was identified for all the nodes in the network. CC is defined as the inverse of the average length of the shortest paths to/from all the other vertices in the graph. The topological centrality of the hubs and the bottlenecks in the networks were inspected by measuring the CC values of the protein set with either large BC value or degree [43]. The shortest path (geodesics) was evaluated by calculating the length of all the geodesics to or from the vertices in the network. The average shortest path was evaluated to analyze the number of average steps required to link the two randomly selected nodes in the network [45]. The nodes obtained as a result were the nodes with a large BC value, a large degree, and both large BC and degree and CC value. These values were analyzed and calculated by the R package.

### 3.4. Construction of a Small PPI Network with Important Nodes

To show simplified main pathways and major proteins, the identified important nodes and interconnected proteins that make a link between them were extracted from the whole network by using Cytoscape.

### 3.5. GO Enrichment Analysis

The ontologies and its genes from the simplified azoospermia protein-protein interaction network was identified using BiNGO plugin for the Cytoscape which are: molecular functions, biological processes, and cellular components [46,47]. For a complete view of the studied biological process, the ClueGo plugin of Cytoscape 3 allows us to integrate several ontology sources because in each source, for each gene, there is a large amount of information. ClueGo can extract the non-redundant biological information for the large cluster of genes, using GO, KEGG, BioCarta, REACTOME and WikiPathways [48].

### 3.6. DAVID v6.7 Analysis

DAVID v6.7 (available on: http://david.abcc.ncifcrf.gov/conversion.jsp) is a program which is available on the web and provides a complete set of functional explanation tools which helps in the understanding of biological meaning of large sets of data of proteins and genes [49]. For the recognition of disease pathways, the uniprot IDs of the associated proteins were submitted to the DAVID [50].

## 4. Conclusions

The genes associated with azoospermia have been extracted from OMIM databases, and in order to construct the PPI network, the protein-protein interactions of the collected proteins have been identified using the Human Protein Reference Database. The built protein interaction network has been analyzed topologically and it is similar to the other networks following a scale-free distribution. The examination of highly connected and bottleneck proteins as key nodes in a scale-free network has expressed that *CREBBP* and *BCAR1* are new candidate genes. These two genes are supposed to be associated with azoospermia by interacting with the key proteins in cellular signaling. We demonstrated that some common pathways in azoospermia are linked with certain types of cancer such as colorectal, pancreatic, chronic myeloid leukemia and prostate cancer. Our analysis also showed that there is a possible genetic connection between liver disease and azoospermia. Further experimental studies will be required to confirm the importance of these new associated genes and the genetic links between azoospermia and the associated diseases. These results, if validated, can be helpful for further infertility research.

## Figures and Tables

**Table 1:** Hub nodes and large BC nodes.

Parameters	Gene Name
(Hub + Large BC) nodes	*AR*, *DAZAP2*, *ESR1*
Hub nodes	*PRMT1*, *ATXN1*, *UBB*, *KHDRBS3*, *DAZ1*, *RBM9*, *PUM2*, *QKI*, *EGFR*
Large BC nodes	*RB1*, *NR3C1*, *SMAD3*, *EP300*, *JUN*, *HDAC1*, *SMAD4*, *MAPK1*, *SP1*, *CREBBP*, *BCRA1*, *RELA*

The highly connected nodes known as hubs are more important than lesser connected nodes and the nodes with large Betweenness Centrality (BC) values are bottleneck proteins within a scale-free network; *AR*, *DAZAP2*, and *ESR1* are hub nodes that have a large BC.

## Supplementary Materials

Supplementary materials can be found at www.mdpi.com/1422-0067/17/11/1857/s1.

## Abbreviations

AR	Androgen Receptor
BC	Betweenness Centrality
CC	Closeness Centrality
DAZAP2	Deleted in azoospermia associated protein 2
ESR1	Estrogen Receptor
GNRH	Gonadotropin-releasing hormone
HPRD	Human Protein Reference Database
ICSI	intracytoplasmic sperm injection
NOA	Non-obstructive azoospermia
OMIM	Online Mendelian Inheritance in Man
PPI	protein–protein interaction
SSC	spermatogonial stem cell
